# *“You talk about problems until you feel free”*: South African adolescent girls’ and young women’s narratives on the value of HIV prevention peer support clubs

**DOI:** 10.1186/s12889-020-09115-4

**Published:** 2020-06-26

**Authors:** Deborah Baron, Fiona Scorgie, Lethabo Ramskin, Nomhle Khoza, Jennifer Schutzman, Anne Stangl, Sheila Harvey, Sinead Delany-Moretlwe, for the EMPOWER study team

**Affiliations:** 1 Wits RHI, Faculty of Health Sciences, University of the Witwatersrand, 22 Esselen Street, Hillbrow, Johannesburg, 2001 South Africa; 2 International Center for Research on Women, Washington, DC USA; 3 London School of Hygiene and Tropical Medicine (LSHTM), London, UK; 4 Mwanza Intervention Trials Unit (MITU), National Institute for Medical Research, Mwanza, Tanzania

**Keywords:** HIV prevention, Clubs, PrEP, Young women, Africa, Empowerment

## Abstract

**Background:**

Daily oral pre-exposure prophylaxis (PrEP) can reduce HIV infection in adolescent girls and young women if used consistently during periods of risk. The EMPOWER study evaluated peer-based clubs incorporating an empowerment curriculum offered to adolescent girls and young women (16–24 years) in South Africa and Tanzania for adherence support.

**Methods:**

Using serial in-depth interviews (*n* = 33), we assessed the benefits and challenges of club attendance among 13 EMPOWER participants in the Johannesburg site who were randomised to clubs. We used a summary matrix of coded data to support a narrative, case-based analysis. Four case studies are presented.

**Results:**

Club participants reported benefits such as increased self-esteem and self-efficacy, reduced isolation, and greater insight into gender-based violence and strategies to address it. Day-to-day PrEP adherence was not the only topic discussed in clubs; participants also appreciated the safe space for sharing problems (such as relationship conflict and PrEP stigma) and found interactive exercises helpful in improving partner communication.

**Conclusions:**

Findings support the use of peer-based clubs using a structured empowerment approach, which may offer valuable PrEP initiation support to adolescent girls and young women in settings with high HIV and gender-based violence prevalence.

**Trial registration:**

Pan African Clinical Trials Registry PACTR202006754762723, 5 April  2020, retrospectively registered.

## Background

With more young people in the world today than ever before, roughly 1.2 billion adolescents aged between 10- and 19-years account for 17% of the 7.2 billion global population worldwide [1]. Facing multiple co-occurring life transitions compounded by enduring restrictive gender norms, adolescents—and especially young women—are at increased risk for HIV [2–4]. According to UNAIDS, adolescent girls and young women (AGYW) aged 15–24 years comprised 19% of new HIV infections in 2017 —of which 80% were living in sub-Saharan Africa [3]. This gender disparity is particularly striking in South Africa, where the HIV prevalence of AGYW aged 15–24 years old is four times higher than males of the same age [5]. While significant advances have been made in the expansion of HIV treatment across southern and Eastern Africa, HIV incidence remains unacceptably high among young African women [6].

Oral pre-exposure prophylaxis (PrEP) offers AGYW a promising prevention method they can initiate and control to reduce their risk of HIV acquisition [7, 8]. Taken as a daily pill optimally at the same time each day, PrEP offers women a safe option for preventing HIV during periods in their life when they may experience regular exposure (e.g. unprotected sex with a partner of unknown or seropositive status). PrEP demonstration projects in South Africa highlight that young women have high interest in starting PrEP, yet early drop-off rates, between 30 and 60% within the first 3 months, reveal significant challenges in their ability to maintain use [9–12]. In addition to the self-efficacy essential for behaviour change (i.e. establishing a daily habit of pill-taking and managing start-up symptoms) [11, 12], AGYW must also navigate gendered social, economic and structural inequalities that create barriers to ongoing and consistent PrEP use. Among others, these barriers include unequal decision-making power in sexual relationships [13], lack of social support [14], depression [15], and community perceptions and social norms that give rise to PrEP stigma [14, 16].

Pioneered in HIV treatment, community-based anti-retroviral treatment (ART) adherence clubs offer peer support in a group format and have been used successfully in South Africa and Mozambique to build the capacity of individuals to adhere to their medication and remain in care [17–19]. Peer clubs are recommended in particular for adolescents, who may be especially responsive to peer influence [20]. This model has been adapted for use in HIV prevention trials, most notably in the FACTS 001 tenofovir gel trial in nine sites across South Africa [21]. The trial used clubs to help the young women tackle immediate adherence challenges, such as managing side effects, and addressed underlying socio-structural barriers to gel use.

One such barrier was pervasive gender-based violence (GBV) in these communities, which has been shown to limit HIV prevention behaviours [22, 23]. Drawing on the FACTS 001 experience, the EMPOWER (Enhancing Methods of Prevention and Options for Women Exposed to Risk) study (DOH-27-0118-5353) developed and evaluated peer-based clubs incorporating an empowerment curriculum. Evidence suggests that group-based participatory violence prevention interventions are effective in addressing underlying social norms that condone violence, and in developing communication and conflict resolution skills within partnerships [24]. Evaluations of this approach in three GBV programs tested in randomised controlled trials in South Africa showed that they helped participants develop greater relationship control, reduce unprotected sex, and develop more equitable gender norms, among other positive outcomes [25–27]. Building on this model, the EMPOWER clubs aimed to increase empowerment and improve partner communication thus supplementing the standard individual-level adherence support AGYWs received in the intervention, by strengthening their communication and conflict resolution skills and supporting the safe introduction of PrEP within sexual partnerships.

To better understand the role and possible benefits of these clubs for AGYW taking PrEP, we undertook a qualitative investigation among a sample of participants attending the clubs in one of the study sites. We examined perceived benefits and challenges to attending the clubs as well as how peer engagement during club sessions affected AGYW’s perceptions of gender norms, relationship dynamics with their sexual partners, and PrEP use.

## Methods

### Overview of the EMPOWER study

EMPOWER offered PrEP as part of a combination HIV prevention package that also addressed GBV and stigma. A total of 431 AGYW aged 16–24 years were enrolled and followed across two sites, one in South Africa and one in Tanzania between August 2016 and February 2018 [28]. The study assessed whether AGYW randomised to receive a standard adherence package (individual counselling and text messages) in addition to being invited to participate in peer-led and activity-based empowerment clubs had better daily PrEP continuation and retention in the program when compared with AGYW who received the standard adherence support alone (i.e. no clubs). Community dialogues with local stakeholders and quarterly retention events, some of which included male partners of study participants, were used to raise awareness about the study, PrEP and HIV prevention broadly. The main findings, reported in detail elsewhere [28], showed there was no statistical difference in PrEP continuation at six months between women randomised to attend clubs and women randomised to receive the standard support package only.

### EMPOWER clubs

To develop the club intervention in EMPOWER, we undertook a literature review of existing empowerment curricula [17, 26, 27, 29, 30] and assessed how they had been used. This review informed our content creation, adapted to reflect the local context, youth cultures, and social and economic realities of young African women. The resulting curriculum was premised on addressing underlying gender norms, including expectations of male and female gender roles and behaviour [31, 32], and focused on building skills for improved communication and conflict resolution using techniques of critical reflection, discussion, and practice [33, 34]. Activities such as interactive role-playing and scenario analysis were developed as opportunities to practice communication and negotiation skills. The final curriculum comprised of four 90-min sessions that EMPOWER study participants could attend once a month on average, designed so that cohort groups moved through the four sessions together. The sessions were structured around inter-linking themes, delivered in the following order: 1) Gender Roles and Social Norms; 2) Power and Control; 3) Sexual and Reproductive Health and 4) Empowerment. Additional open-ended club sessions were offered monthly for continued support following conclusion of the curriculum.

### Qualitative study

#### Sampling and recruitment

Between February 2017 and February 2018, we conducted serial individual in-depth interviews (IDIs) with a sub-sample of EMPOWER study participants in Johannesburg (*n* = 25) to assess the implementation, acceptability and feasibility of the study interventions [28].

Qualitative participants were sampled in proportion to the ratio of PrEP acceptors and decliners in the main EMPOWER study, and included women randomised to both intervention and control arms. Mid-way through the recruitment process, the sampling strategy was made more purposive to recruit women with a broader range of experiences, including those who had stopped PrEP use and women who had experienced GBV.

#### Study setting

Participants were recruited from clinics, nearby college campuses and the community surrounding them in inner city Johannesburg. Hillbrow is a high-density inner city residential neighbourhood with a large migrant population, including both economic migrants from across the country as well as immigrants and refugees from the continent of Africa more broadly. Accommodation in the area is in short supply, with residents living in over-crowded high-rise apartments, many of which are in a state of decay and experience frequent interruptions to power and water supply [35]. In the post-apartheid era, there has been little public sector investment in Hillbrow and neighbouring suburbs of the inner city, and urban infrastructure is poorly maintained [36]. More than a third of the inner city’s resident population is estimated to be unemployed [37], and many residents make a meagre living from small-scale informal trading, domestic work, and mobile cross-border trading [36].

#### Data collection

We made use of serial individual in-depth interviewing (repeat interviews with the same participant over time) to collect the data. Each participant was interviewed between two and three times over the 13-month duration of the qualitative study. Detailed IDI guides were developed, and two interviewers were trained in their use, one of whom had also facilitated club sessions. After the first round of interviews was completed, the guides were adapted to include new questions and refine probes, based on the content of emerging data (see Table 1).

**Table 1:** Key Interview Guide Topic Areas Across Time Points

Interview # (Time point)	Key Areas / Focus of Interview Questions
Interview 1 (Month 3)	• Motivations for PrEP uptake
	• Initial experiences and challenges with taking PrEP
	• Disclosure of PrEP use and/or study participation to partners, family and broader network of friends
Interview 2 (Month 6)	• Life changes (relationship status, living/work/school situation, PrEP use, health status, etc.) since previous interview
	• Influences on ability to continue PrEP use
	• Experiences with study interventions (e.g. clubs, adherence support, referrals)
	• Perspectives on community perceptions of PrEP
Interview 3 (Month 9, 12 or Exit Visit)	• Life changes (relationship status, living/work/school situation, PrEP use, health status, etc.) since previous interview
	• Perspectives on study interventions, including participation in clubs for those randomised to this group
	• Overall experiences of study participation, including PrEP use

Each interview lasted between 35 and 70 min and was carried out in the participant’s preferred language (mainly English, isiZulu, or seSotho). Interviews were audio-recorded, transcribed and translated into English, where necessary. Transcripts were checked against the audio recording for quality checks before being uploaded to a central, secure database and imported into NVIVO 11 for analysis.

#### Data analysis

Interpretations of the emerging data were discussed, and specific themes identified in two analysis workshops. Following open coding of a small selection of transcripts by six members of the qualitative team, a codebook was developed with first-cycle topical codes (e.g. HIV risk, clubs, PrEP motivation) that closely aligned to themes in the interview guides. The codebook was then refined and used by four authors to code the full dataset.

Of the 25 participants interviewed in Johannesburg, 13 had been randomised to participate in the EMPOWER clubs. Additional analysis was undertaken on the transcripts (*n* = 33) of these 13 women. A complementary codebook was developed to analyse data on club participation specifically, focusing on perceived benefits, dislikes and challenges relating to women’s participation in the clubs, as well as on changes in attitudes and behaviours as a result of club participation. A separate narrative analysis [38] was undertaken to highlight unique stories of life changes, which are made visible through use of longitudinal data [39]. To facilitate further analysis of patterns, comparisons and themes within and between the participants’ narratives, a time-order summary matrix was compiled [40]. This matrix enabled dominant themes to be analysed in concert with participants’ responses to direct questions about changes in PrEP use, sexual relationships, risk behaviours, and overall life circumstances since their previous interview. Using a narrative, case-based approach allowed us to analyse how the clubs became significant and meaningful to women not only for PrEP use and HIV prevention specifically, but also within the larger context of their lives.

#### Ethical approval

Ethical approval for the study was obtained from the Human Research Ethics Committee of University of the Witwatersrand in Johannesburg, South Africa.

## Results

### Demographic characteristics

The 13 participants included in this qualitative analysis ranged in age from 18 to 24 years; the median age was 21 years (IQR 18.5–22). More than two-thirds (9/13) were tertiary-level students in the inner city attending nearby universities or technical colleges, with the remainder having completed high school and seeking employment. All were living with parents, other relatives or in student residences. Prevalence of GBV was high, with 60.5% (8/13) reporting ever having experienced physical, sexual or emotional violence, compared to the 52% who screened positive for lifetime GBV at enrolment in the Johannesburg qualitative sample as a whole (see Table 2).

**Table 2:** Demographic, PrEP Use and Club Participation Characteristics (Listed in order of number of club sessions attended; least to most)

Participant pseudonym, Age range in years	No. of clubs attended	Education/ Employ-ment	Relationship status at start of study	Life-time GBV Experience^a^	PrEP acceptor at first visit (yes/delayed)	Self-reported PrEP use and challenges^b^	PrEP continuation to study end^c^
Olive, 21–24	0	University student	Partner of > 1 year	Yes	Yes	Generally used PrEP consistently. Missed doses when away from home.	Maintained PrEP use.
Mbali, 21–24	0	University student	Partner of > 1 year	Yes	Yes	PrEP use became easier following disclosure to partner, who was controlling but accepted her use of PrEP.	Maintained PrEP use.
Samu, 18–20	2	University student	Single	Yes	Delayed due to mother’s opposition; became covert user	Inconsistent PrEP use throughout study, especially during periods of sexual abstinence.	Maintained PrEP use.
Lerato, 18–20	2	University student	Partner of < 1 year; separate lover of > 1 year	Yes	Yes	Consistent PrEP use despite lack of support from partner.	Temporarily discontinued with intention to re-start.
Prudence, 18–20	3	University student	Partner of > 1 year	No	Yes	Inconsistent PrEP use and relied on partner to remind her.	Discontinued use when relationship ended.
Pamela, 18–20	4	High school student	Partner of > 1 year	No	Yes	Generally used PrEP consistently. Missed doses when routine changed unexpectedly.	Maintained PrEP use.
Busi, 21–24	4	Completed high school; employed	Partner of > 1 year	Yes	Yes	Generally used PrEP consistently when in relationship. Noted no adherence challenges.	Discontinued use when relationship ended, and risk perceived to be zero. Distance to clinic considered a burden.
Mariam, 18–20	5	Completed high school; employed	Partner of < 1 year	No	Yes	Consistent PrEP use improved over time as usage became more routine.	Maintained PrEP use.
Zanele, 21–24	5	University student	Partner of < 1 year	No	Yes	Inconsistent PrEP use.	Discontinued use when decided to get pregnant. HIV sero-converted a few months after discontinuation.
Thuli, 21–24	5	University student	Partner of > 1 year	Yes	Yes	Inconsistent PrEP use. Wanted a long-acting option.	Maintained PrEP use.
Dudu, 21–24	5	Completed high school; employed	Partner of < 1 year	No	Yes	Consistent PrEP use.	Temporarily discontinued use when became pregnant. Intention to re-start.
Nelisiwe, 18–20	6	Completed high school; employed	Partner of < 1 year	Yes	Yes	Initially an inconsistent PrEP user. Consistent PrEP use improved after first few months.	Maintained PrEP use.
Lillian, 21–24	6	University student	Partner of < 1 year	Yes	Yes	Generally consistent PrEP use. Missed doses when routine changed unexpectedly.	Temporarily discontinued with intention to re-start.

^a^ Our definition of GBV encompasses intimate partner violence (IPV) and also any violence (physical, emotional, sexual and economic) perpetrated by family members, peers and strangers. Includes both past and current experiences

^b^ Data extracted from IDIs and presented as self-reported by participants, not objectively measured

^c^ Refers to time frame of study participation, varying from 12 to 18 months

All but one were in a relationship with a primary partner, and about half reported inconsistent condom use. Of the 12 women with a regular sexual partner, 11 described their partners negatively, as either “*controlling*”, “*cheating*” or “*lying*”. While all 13 participants had accepted PrEP at or shortly after enrolment, by study completion, only seven had maintained PrEP use throughout their study participation (which ranged from 5.8 to 15.3 months). Three had temporarily stopped PrEP due to a life transition (such as pregnancy or ending a long-term relationship) yet aimed to re-start use in the imminent future. The remaining three participants had discontinued PrEP with no intention of resuming use.

### Experiences of club sessions

Interview participants had attended between zero and six club sessions. Overall, 11 of the 13 interview participants attended at least one club session, compared with 50% of the overall study cohort randomised to clubs. Eight interviewees attended all four sessions of the curriculum (compared to 18% of the total study cohort), of which six continued coming to sessions even when the curriculum was complete, attending five to six club sessions in total [28]. Each session included between two and ten women. Making time to attend club sessions was challenging as most participants had classes and other obligations during the day. Club sessions were generally scheduled to coincide with study clinic visits, thereby reducing the visit schedule burden. However, participants complained that this resulted in longer visits to the clinic than anticipated. Saturday club sessions were also introduced and scheduled strategically to align with monthly social calendar norms (i.e. avoid pay-cheque weekends), which were better attended.

Despite the clubs being designed so that cohort groups would move through the four sessions together, some women swapped groups due to scheduling conflicts. These changes appeared to undermine group cohesion and create anxiety for some women who preferred to remain with their original group, especially when rapport, trust and friendships had been established. For example, Busi, a young working mother, was dismayed when a new work schedule forced her to change her club group.

*So it was awkward for us to feel… for me to feel free because I was not used to those girls because obviously if we know each other from way back, it’s easy for us to talk.*

Switching groups meant there was a need to re-establish peer relationships and re-build trust each time. Some women chose to simply arrive late if they had scheduling conflicts or experienced challenges with public transport to the clinic. Lack of punctuality and poor attendance were cited as a key dislike by those who did arrive on time. At least one participant described being the only participant to arrive for a session 1 day, and the session subsequently being cancelled.

Overall, the women who did attend sessions said they liked both the content and the interactive format. One participant, Dudu, captured a common sentiment, highlighting the clubs’ provision of a safe space for sharing problems, saying:

*Like, the club meetings help us, like, we are free to talk about anything, like, any problems that we…So we are like friends, like, we feel free to talk about anything because we know that we don’t know each other… You know that your life and stuff, like, they are safe with them, so I think they [clubs] are very good.*

While clubs were generally facilitated by a peer-aged coordinator, the session that focused on GBV required additional psycho-social support, which was provided by older trained counsellors who co-facilitated these sessions. Most participants saw value in having them present. Reflecting a broader cultural norm in South Africa, in which young people are expected to defer to and learn from their elders, Lillian explained how younger club participants could draw on the experiences and life expertise of older women in the group:

*…they [older women] give us the advice to say, hey, ‘if this happens, this happens’. Because they have been through those [experiences], so I think it’s fine, the old ones should continue, isn’t it, they say ‘****indlela ibuzwa kwabaphambili’****[a Zulu idiom which means ‘you ask for directions from those who have gone past it’], ja.*

Despite logistical and scheduling challenges, most of those who managed to attend club sessions reported finding them personally transformative. In the sections that follow, we unpack the nature of these transformations through case studies of four of the 13 participants, highlighting key aspects of their narratives over time. To select these cases, we created a conceptual grid juxtaposing patterns of PrEP use with club attendance (see Table 3) and allocated the 13 participants assigned to attend clubs to this grid. Individual cases representing the different fields of this grid were then selected to capture a broad spread of experiences.

**Table 3:** Participants by PrEP Continuation & Club Participation

CLUB PARTICIPATION (attended at least 1 session)	PrEP USE		
	Uninterrupted throughout study	Paused but intended to restart when needed	Discontinued during study
Yes	**Neli**^**a**^	**Lerato**^**a**^	**Zanele**^**a**^
	Thuli	Dudu	Busi
	Samu	Lilian	Prudence
	Pamela		
	Mariam		
No	**Olive**^**a**^	–	–
	Mbali		

^a^Participant pseudonyms in bold are participants presented in the case studies

### The value of social support: the case of Neli

Studying to re-take her final school exams, Neli spent most of her time moving between the confined spaces of school and her parents’ separate homes. Fearful that her PrEP use would inadvertently expose her as sexually active, Neli hid her PrEP and reasons for going to the clinic from her mother, citing headaches or stomach pains instead. Despite her mother’s strictness, she had managed to maintain a secret relationship for over a year. Neli characterised her relationship dynamics before joining EMPOWER as:

*…unfair because it was one-sided...like it was more like whatever he says should stand whether I disagree or I agree. It’s [like] my word did not count.*

She summed him up as *“controlling in a nice way”*, stating he was never physically violent toward her. She took precautions to prevent any provocation, however, also concealing her PrEP use from him, saying *“I’m avoiding many problems.”*

Like many women in the study, Neli was strongly motivated to start PrEP because she recognised her acute risk for HIV infection. Condoms were not used with her partner and she did not trust him to be faithful to her. She used contraception to prevent pregnancy, and now PrEP would protect her from HIV. But adherence was challenging, and those early days required strong self-motivation and discipline. She had little pill-taking experience and in fact disliked taking pills altogether. Having overcome the initial side effects of dizziness and nausea, she did not want anyone to dissuade her from continuing PrEP use. As an early PrEP adopter, Neli was therefore cautious about disclosing to others in her community, recognising that *“the majority are not aware of the PrEP pill”*. She worried about potential stigma attached to PrEP use, and during her first interview, she imagined how people might respond:

*‘Why is she using PrEP? It means she is sleeping around’. [And so] for me I feel, when somebody judges me I feel discouraged. I wouldn’t want--, if it’s just the group [club peers] that knows, it’s okay for me.*

In this way, Neli’s club peers were the only people who knew of her PrEP use. Not only did they share similar motivations for using it, they also became a critical source of emotional support over time.

*Well, [takes a deep breath] it’s like a girls’ talk, you know where you get to be open, when you get to hear of other people’s views, neh? That is where you got to talk about how you feel and it’s very helpful to those whom are mainly discouraged.*

Neli further valued club sessions for the content of the curriculum, recalling *“we learnt about confidence, we learnt about esteem, we learnt about gender-based violence. We learnt a lot.”* Not surprisingly, Neli attended nearly every club she could, five out of six in all.

When she returned for her second interview four months later, she spoke about how the new knowledge had become integrated into her life. She talked extensively about the positive impact of clubs on her self-confidence and on her relationship with her partner.

*[S]ince from the last time I came here, a lot of things have changed and I feel I began to have the self-esteem to stand firm in whatever I want to do in the relationship specifically….I had problems, I couldn’t speak up, you know, I couldn’t express how I feel….But ever since I came here, I was able to challenge the world, I was able to face whatever and express myself on how I feel, ja.*

Immersing herself in the stories of other women in the clubs also helped shift Neli’s beliefs about GBV.

*Well, I was at first, when it came to gender-based violence, I was more into [believing] if the woman was wrong, then she deserves the punishment. If the guy is wrong so, so, he also deserves the punishment. But since coming here and hearing other people’s views and stuff, I understood that gender violence is bad, it’s never good… It is not that somebody has to be wrong or right, it is itself just wrong and it should be reported if there is any sign of gender violence in any environment or community.*

As the clubs influenced her attitudes about gender and power in relationships, things began to change in her own relationship.

*From the groups and from the clubs that we’ve been attending I learnt to stand firm and always have a word in everything that is happening in a relationship. So eventually things started changing, we started understanding each other and it was now both-sided and not one-sided. So ja, things are now controllable.*

By the second interview, Neli had settled into a daily adherence routine, although she continued to take her PrEP pills covertly. Having chosen not to disclose PrEP use to anyone, club participation appears to have filled a gap that otherwise may have been provided by supportive family members or a partner. It also appears to have ignited a new-found consciousness and confidence for Neli, enabling other positive transformations in her life. When asked whether she would attend clubs in the future if convened at public clinics, Neli replied, *“I would, I would be the first one and I’m sure I would be in the front seat.”*

### Motivation to exit an unhealthy relationship: the case of Lerato

Having started dating early in life, Lerato had been with her partner since the age of 13. She was aware that his regular infidelities placed her at risk, but nonetheless acknowledged that she “was that person who didn’t use condoms like at all.” In addition, his cheating had led her to take a “side lover” as well, a fellow student where she attended university, who himself had another concurrent girlfriend. During Lerato’s first interview, she mentioned that her partner had hit her once and she feared further violence. He did not support her PrEP use either, saying he “did not take the pill seriously”.

During her second interview, she revealed that she had not understood the difference between the clinic visits and club sessions—and hence hadn’t attended any sessions. As a busy student, she was also concerned about the extra time commitment being asked of her, saying *“Hey, I am busy, like one main reason that makes me not to come here is because you take time, guys.”* But there were other reasons Lerato had missed club sessions. During this same interview, she also revealed that her partner had beaten her after finding out about her side lover. She shared:

*I told you I couldn’t come [to the club] because I was messed up, do you remember? I had a blue eye [laughs] ...and I was writing exams.*

After this beating, Lerato decided to end the affair. She revealed that her partner had agreed to refrain from hitting her again, provided she behaved as *“a good girl”.* Indeed, she claimed to be doing just that: *“I am not sleeping with many people, I have one sexual partner.”*

Between the second and final interview, Lerato experienced several important changes in her life. She had begun to attend club sessions and to reflect more critically on the relationship with her partner. At the first session she attended, which focused on GBV, she described arguing with some of her club peers, noting:

*…they had their own views and I had my own views so, ja, we were in a different world ‘cos we didn’t have anything in common…I felt like girls let their men rule their relationships, they don’t know how to stand for themselves. ‘Cos I believe that when you have complaints in a relationship, it’s your choice to stay or go. If you prefer to stay, you have to be able to face the challenges. So they are complaining but they are still staying. So I didn’t get that.*

Lerato herself eventually made the decision to *“stand for”* herself. After discovering that her partner had cheated on her yet again, she ended the relationship. She later reflected that club participation had given her pause for thought about her initial impatience with her peers who were struggling with unhappy relationships:

*I learnt that every relationship has its own way and strategy they use to last, so ja, so, I for one should learn not to judge other relationships ‘cos I judge a lot, so ja….*

Critically, this shift in perspective appears to have been enabled by the supportive environment of the clubs and her overall study participation. Referring to her own experience of GBV, Lerato said:

*In a way, the study made me open up, talk more about what happened in my relationship, it helped, ‘cos social workers were here to assist us, talk to us about what had happened, who we could contact for help.*

Following the break-up with her partner, she decided to stop PrEP, and planned to re-start it when she became sexually active again. Hopeful for a new relationship in the future, Lerato credited the study and the two club sessions she had attended with helping to empower her to take charge of her health and her life more broadly.

*[They have] made me take my health seriously ‘cos I started preventing, taking PrEP, attending studies, learning more about what other women face, how we could conquer all problems and come up with strategies to make our men listen to us and our needs.*

Importantly, she described how the sessions had been useful toward building practical strategies for dealing with PrEP disclosure in future relationships:

*“we were even [role-play] acting…We came up with ways to make--, to apply communication on relationships to explain PrEP to other people and understand and ways in which you can explain to people who don’t want to understand, so, ja, it was helpful."*

These role-play activities may have helped participants like Lerato communicate their HIV prevention needs with their sexual partners and take ‘ownership’ of it – just as they enabled her to claim her right to be in a non-abusive relationship. Moreover, being able to ‘explain to people who don’t want to understand’ is a critical part of raising awareness at community level about—and acceptance of—PrEP as an HIV prevention strategy for young women.

### When clubs alone are not enough: the case of Zanele

At the time of her first interview, Zanele, a university student, had a long-term partner with whom condoms were not used even though she did not trust him. When asked why she wanted to use PrEP, Zanele’s story was like so many of the young women in EMPOWER. Put simply, she wanted to participate:

*…because eish, I can’t trust my boyfriend…especially if you are sexually active. Then so when I looked at it, indeed it was in line with my situation, to say okay I am sexually-active and I am not using condom, I am at risk of getting HIV ‘cos I don’t even trust my boyfriend.*

Zanele initially only disclosed her PrEP use to her younger female relatives and encouraged her cousins to also join the study. Her older sister disapproved, however, and reprimanded Zanele by saying, “*people who take PrEP are those who have multiple partners*”. Zanele refrained from telling any other family members for fear of attracting additional judgement or stigma. Like Neli, she also strategically did not disclose to her partner, fearing that if she told him, “*a fight will start, World War 3 will start*.” Zanele’s anticipated disapproval from her partner, which she feared could even result in physical violence, reflected similar concerns expressed by several participants in the study.

At Zanele’s second interview, she shared that her cousins had since joined another HIV prevention clinical trial near their home in Soweto. Possibly emboldened by this new information, Zanele decided to tell her mother about her own participation in EMPOWER. Her mom then began to support her, by reminding her to take her daily pill – referring to PrEP in code as “*that thing*” in order to avoid Zanele’s father finding out about it. Still, Zanele had chosen not to disclose to her partner, partly because they were beginning to drift apart in their relationship, as they “*focused on external things.*” She was optimistic throughout this interview though, confidently noting:

*You know, I think being part of this study helped me make wise decisions, I think about everything…everything has changed.*

Speaking of the clubs, Zanele said, “*I found people who are loving and welcoming,*” which brought out a new side to her. She claimed her partner “*would be shocked*” to see her during a club session “*because I am just a quiet person, but I have just found that platform where I can just also express myself*.” She, too, found value in the club curriculum, and described the empowering effect of acquiring new knowledge in a safe, mediated space.

*You see these topics that we get, for me they are okay because in a way because we talk about them, something comes out and then we again unpack them, ja, just anything….like you get many ideas, and in way, eh, amongst those ideas that you get, you can put all of them together and make a better decision for yourself.*

Zanele felt that even though she had not personally experienced GBV, exploring this issue in the clubs had helped to raise her consciousness about the power dynamics in her own relationship and how to navigate them better. She noted:

*Okay, in terms of relationships, eh, I have learned that communication is key. You see, when you communicate, you do not do something just because another person wants you to do it, you both need to take decisions as a couple, you need to be responsible for those decisions, there was this other time, I cannot recall what the topic was, we were talking about rights and responsibilities.*

In contrast to the hopefulness expressed in her second interview, Zanele’s final interview was filled with sadness and regret. In early September, unaware that PrEP does not interfere with conception, she decided of her own accord to stop using PrEP following a decision taken with her partner to have a baby. Shortly thereafter, she went to the clinic for her quarterly visit, and reported, “*Ehh, only found that on my last visit, the 22nd of November, ehh, I was tested HIV positive.”* Zanele cried during the interview as she spoke about how she regretted stopping PrEP, saying, *“I no longer trusted him since he had changed. If I had listened to my instincts I wouldn’t be here now.”* Following her diagnosis, she had disclosed her HIV-status to her partner and revealed the entire chronology of her participation in the EMPOWER study and use of PrEP. He blamed her for bringing HIV into the relationship and they broke up.

Zanele was not ready to tell anyone in her family about her seroconversion. The only other people she had confided in were peers in the EMPOWER clubs, “*Ja, strangers that I have met here in the study so far”*. In this respect, the clubs functioned as a safe haven. At first, they had provided a protected space away from her untrustworthy partner, where she could freely express a new-found confidence. And when circumstances and prevailing social pressures surpassed her best intentions to make healthy, positive choices, her club peers were there to offer non-judgmental support. Importantly, she revealed how she had urged them to continue PrEP if they did not want to end up like her, telling them, *“guys, you need to take it every day. It works. Take it every day.”* In short, sharing her own experience as a cautionary tale became Zanele’s way to reciprocate the support she had received over time from attending the clubs.

### Club attendance sabotaged by controlling partners: the case of Olive

Olive was one of only two women in the sub-sample who did not attend any of the club sessions. Despite being randomised to this group and receiving information about the clubs and regular reminder text messages, both women said they knew little to nothing about how the clubs worked, or the content and focus of these clubs.

For Olive, a college student who lived with her partner of one-year, studying full-time meant there was simply no time to attend club sessions. During her final interview, she revealed that her partner also had discouraged her attendance, noting “*Sometimes, like, that person used to stop me from coming*.” Olive described her partner as controlling and emotionally abusive, but tolerated his behaviour given her financial dependency on him. During her first interview, she openly admitted to not liking him, but rationalised that:

*Okay, like he is my provider now, he provides for me and accommodation, ja, I don’t have a place to go to so that I can go to school, so I have to stay with him, I have to face the situation, I had to do whatever he wants.*

Olive described how the relationship had become increasingly negative, especially once she had disclosed that she was using PrEP. Her partner questioned this, accused her of being HIV-positive, and obstructed her PrEP use:

*…he would, like, take my pills and hide them from me…But when he goes to work the following day, I would hunt them and find them…he said that he doesn’t believe that this thing, he doesn’t trust Truvada.*

Olive told friends about this increasingly abusive situation, but they had “*nothing*” to say in response. She then went to the clinic to get another HIV test and showed him her HIV test results in an effort to prove her sero-negative status, but he ceased all sexual relations with her nonetheless. Stating, “*I know that I like sex very much*”, Olive then started having sex with other men, rationalising this as “*I did that just because he was not sleeping with me…[but] I would use protection*.”

During Olive’s second interview five months later, she shared that her partner had broken up with her. He had told her to leave his place, forcing her to find alternative housing at short notice. She lived alone now, and her sister paid for her accommodation. She admitted, “*Eish, like I feel so relieved, like, I feel like I was put in jail there*.”

When the interviewer explained what the club sessions had covered and encouraged her to join the remaining sessions, Olive expressed regret. Realising that the clubs may have helped her stand up to her controlling partner especially when disclosing PrEP use to him, she said, “*I have seen that I have missed a lot*.” Olive’s absence from the clubs did not seem to impact negatively on her PrEP use, however. She remained fully committed to using PrEP throughout the study and continued to take calculated risks about when to also use condoms and when to rely solely on PrEP to protect herself from HIV.

## Discussion

Peer-based support clubs have been used worldwide to assist people dealing with a range of illnesses and social issues, from addiction treatment [41] to mental health problems [42] and breast cancer [43]. This study adapted an empowerment-based approach [25, 26] delivered through participatory group-based activities previously shown to be effective in reducing intimate partner violence [27] and supporting ART adherence [17]. Unlike the traditional support club model, however, EMPOWER clubs were designed to meet the needs of a population seeking to *prevent* illness rather than to cope with a new diagnosis. These ‘early adopters’ [44] of a new HIV prevention technology were brought together by a shared motivation to remain HIV-uninfected. Together, the case studies presented here illustrate some of the key benefits these women derived from attending EMPOWER clubs: increased self-esteem and self-efficacy to control one’s destiny, reduced isolation via the creation of social networks, and shifts in attitudes towards more equitable gender norms. Club sessions provided an alternative social context and ‘safe space’ where participants could share intimate details about their sexual relationships and sensitive life events without fear of judgement.

While the clubs provided a valuable platform for peer support that women were often lacking, the club sessions were not accessible to everyone. Our study found that logistical challenges, such as time constraints, transport challenges, and scheduling conflicts, partially accounted for non-attendance. They also had implications for those who *did* attend sessions, straining social cohesion when women could not always remain with the same cohort. Despite these challenges, club attendees largely enjoyed and appreciated the time afforded in the curriculum to deliberate on issues often unspoken and considered taboo in other contexts. Any attempt to roll-out clubs in future PrEP programs will need to address the busy nature of AGYW’s lives, many of whom are juggling school and relationships alongside household and family obligations, and the search for employment.

Across the qualitative sub-sample, women were mostly motivated to start PrEP because of mistrust in their relationships owing to partners’ previous infidelities, and their controlling and sometimes abusive behaviours. However, these same issues also led some women to conceal their PrEP use from sexual partners, fearing disclosure would lead to conflict in their relationship [45]. Frank discussions during club sessions allowed women to weigh their options, and to test out different role-playing disclosure scenarios. Beyond disclosure, the clubs provided an opportunity to practice and strengthen communication skills within intimate partnerships more broadly. This key component of the club curriculum was highlighted by a number of participants as equipping them to make better decisions for themselves and stand their ground with domineering partners.

Because the club intervention in EMPOWER was specifically designed to follow an empowerment curriculum, consistent PrEP use was usually framed within the context of conversations about gender norms, GBV and relationship power dynamics. Of all the topics covered in the club sessions, it was these themes that resonated the most with participants and dominated their narratives. This was perhaps not surprising, as participants with lifetime GBV experience were over-represented in this qualitative sub-sample compared to the overall trial population. In the interviews, many participants described negative relationship experiences, including controlling behaviour and experiences of physical and sexual violence. As shown by the case studies, conversations in club sessions about the reflection of traditional gender norms in personal experiences shifted some women’s thinking in a manner akin to the feminist consciousness raising of the 1960s and 70s [46]. In this process of collective knowledge-production, it was their perspectives on GBV that were most noticeably transformed. Participants shifted from a focus on individual victim-blaming to recognition of violence as a socio-political phenomenon and expression of unequal gendered power relations [32]. Positive impacts were also observed in raised self-esteem, which galvanised some participants into confronting power imbalances in their sexual relationships or exiting these relationships altogether. Similar transformative effects have been noted in other settings where peer-led, curriculum-based sessions using an empowerment approach have been tested. One example is Project LifeSkills for young transgender women in the US, which was found to be highly effective in changing HIV risk behaviours, largely because its structured sessions were “grounded in participant social realities, focused on empowerment and practical needs, and delivered by peers” [47].

When it came to reflecting on the impact of EMPOWER club attendance on PrEP use, we found that participants did not always make the link between their club experience and day-to-day PrEP adherence per se. Rather, they pointed to other elements from the standard adherence package, including the individual-level adherence counselling and text message reminders, as a source of beneficial adherence support. The absence of any substantial commentary in participants’ narratives on how the clubs directly impacted on their own PrEP adherence suggests that this was indeed a critical gap in the club intervention. It may be that the potential for clubs to influence consistency of PrEP use is greater around times when young women are grappling with ‘seasons of risk’ [48] in their lives, and the challenge of aligning PrEP use to these seasons—as we saw clearly in Zanele’s outspoken encouragement of her peers around the time of her seroconversion. While EMPOWER did not find that club attendance improved participants’ PrEP continuation [28], it is worth noting that a separate study conducted in three African cities, including Johannesburg in which two-thirds of participants attended at least one adherence club session, found that attending a club session within the first three months of joining the program was a significant predictor of high (> 4 doses of tenofovir diphosphate per week) versus low (< 4 doses per week) PrEP adherence when measured at month three [49]. Unlike EMPOWER, this study did not include a structured curriculum initially, although this was used later. All participants could attend clubs in that study, and there was no randomised evaluation of club attendance.

There were limitations to our study. Firstly, the study population was limited to AGYW living in a densely populated inner-city area of Johannesburg, South Africa, and the analysis focused on only a small proportion of this sample. Even so, we believe the findings presented here offer useful insights into young women’s experiences of a PrEP support intervention in a context defined by high prevalence of HIV and GBV, and where broad support for a new HIV prevention method is still embryonic. These conditions are true of numerous settings elsewhere in sub-Saharan Africa, suggesting that findings may have relevance beyond this study population and geographical location. Secondly, some of the participant interviews were carried out by an interviewer who had facilitated many of the EMPOWER club sessions. This may have led to social desirability bias, with participants providing more positive and less critical responses than they otherwise might have done in the presence of a neutral interlocutor. On the other hand, the participants may have valued the already established relationship and so may have been more forthcoming in their responses.

The prospect of delivering an HIV prevention package that includes PrEP is both urgent and necessary if we are to stem the tide of new HIV infections in South Africa, especially for AGYW at high risk of HIV. Already, PrEP programs in South Africa designed for young women are establishing partnerships with college campus clinics to provide PrEP to AGYW [50]. These partnerships may provide convenient venues on campuses for university students to attend peer clubs around their class schedule. To reach the most vulnerable AGYW (i.e. not studying or not living in urban centres), alternative club designs may be needed to address logistical and social barriers such as stigma. For example, future programs could consider integrating medication distribution with PrEP clubs, as is currently done in adherence clubs for stable patients living with HIV [51]. In these instances, long-term PrEP users could make use of HIV self-testing, undergo required clinical checks, and collect their next three-month PrEP supply—all while attending a club session. Virtual support groups running on social media platforms (e.g. WhatsApp) may help to circumvent time and transport challenges that prevent face-to-face club attendance [52].

## Conclusions

As countries in sub-Saharan Africa begin to scale up PrEP programs for AGYW, peer support clubs should be considered as a strategy to support consistent use, particularly during periods of high risk. When based on a structured empowerment approach, the club format appears to tap into peer support and helps build resilience during the initial stages of PrEP uptake, when users are grappling most acutely with side effects and decisions about disclosure, and anticipating (or even experiencing) PrEP stigma. For PrEP to be fully incorporated into AGYW’s lives, they require social support that ideally not only addresses immediate adherence challenges – such as remembering to take a daily pill – but also empowers them with tools for greater self-efficacy in a gender inequitable society. After all, it is these inequities, lived most directly in the day-to-day context of sexual relationships, that are so influential in shaping AGYW’s ability to protect themselves from HIV. Clubs may offer a path towards dismantling these structural inequities. They also hold promise as a strategy for empowering AGYW to become advocates for PrEP as a new prevention method in their communities, thereby helping to counter stigma and building a ‘critical mass’ of support for women-centred HIV prevention more broadly.

## Data Availability

The datasets analysed during the current study are available from the corresponding author on reasonable request.

## Abbreviations

AGYW: Adolescent girls and young women
ART: Anti-retroviral treatment
EMPOWER: Enhancing Methods of Prevention and Options for Women Exposed to Risk
GBV: Gender-based violence
HIV: Human Immunodeficiency Virus
IDI: Individual in-depth interviews
PrEP: Pre-exposure prophylaxis
